# Congenital Thrombophilia and Intracardiac Thrombosis: Probably an Underdiagnosed Event

**DOI:** 10.4021/cr278e

**Published:** 2013-07-11

**Authors:** Antonio Girolami, Giulia Berti de Marinis, Martina Treleani, Valentina Tasinato, Bruno Girolami

**Affiliations:** a Department of Medicine, University of Padua, Medical School, Padua, Italy; b Division of General Medicine, Padua City Hospital, Padua, Italy

**Keywords:** Cardiac thrombosis, Thrombophilia, Protein C deficiency, Antithrombin deficiency

## Abstract

**Background:**

To investigate the number of patients with congenital thrombophilia who presented an intracardiac thrombosis.

**Methods:**

Personal files were reevaluated together with a time-unlimited search of the literature.

**Results:**

Twenty-five patients with intracardiac thrombosis and congenital thrombophilia have been gathered from the literature including the two personal cases. The distribution observed in thrombophilia patients is similar for left side or right side heart (9 vs 11 cases). The left ventricle and the right ventricle were involved in six or five instances, respectively. In one case, both ventricles were involved. On the contrary, the left atrium was involved in three cases whereas the right atrium was affected in six cases. In the remaining cases, more than one heart chamber was involved.

**Conclusions:**

In “normal” subjects, left side thromboses are predominant once catheter-associated thrombi are excluded. The reason of this discrepancy lies in the greater prothrombotic effect exercised by congenital thrombophilia on venous thrombosis compared to arterial thrombosis. The relative high prevalence of cardiac thrombosis seen in patients with antithrombin and protein C deficiencies indicated that a cardiac evaluation should be carried out in all patients with these two defects.

## Introduction

Intracardiac thrombosis (ICT) is a relatively rare cardiological condition but in the post-infarction period during atrial fibrillation (AF) and in dilatative cardiomyopathy [1-4]. A rare cause of ICT is represented by congenital prothrombotic or hypercoagulable conditions. Several case reports dealing with ICT and single prothrombotic defects have been presented. The reported cases concerned mainly protein C and protein S deficiencies. However no systematic evaluation on the subject has ever been published. The purpose of the present study was to evaluate all reported cases of ICT occurring in patients with antithrombin (AT), protein C and protein S deficiencies and other known congenital prothrombotic conditions.

## Patients and Methods

Personal files obtained from hospital admissions during the years 1980 - 2006 were reevaluated. A Pub Med extensive analysis using pertinent and multiple key words was carried out without temporal limitations. Original papers were obtained with the help of the Pinali Medical Library of our University. Cross-checking of the references of the single papers was carried out in order to avoid omissions.

Patients with ICT due to a recent myocardial infarction (MI) have been excluded. Cases with dilated cardiomyopathy have also been excluded. All other cases have been included, including two cases with AF. Cases of thrombosis in native or prosthetic valve have also been included and assigned to the atria in the case of mitral or tricuspid valve involvement or to the ventricles in case of aortic or pulmonary valve involvement. The same is true for cases with mural thrombi in the absence of a previous MI. The thrombus had to be demonstrated in all cases by objective means, namely transesophageal echography or angiography. The basal heart condition of the patient together with the presence of associated prothrombotic risk factors (age above 70, infections, heart disease, deep vein thrombosis (DVT), etc…) was recorded. The therapeutic approaches followed were also taken into consideration, together with the evolution of the condition.

## Results

Twenty-five cases of ICT were gathered from the literature (Table 1, 2) [5-29]. Two patients were obtained from personal files (a case of protein S deficiency and another with homozygous FV Leiden) as previously reported [5, 6]. Altogether intracardiac and valvular thrombus have been seen in at least two cases of AT deficiency, in one case of Heparin co-factor II deficiency, in at least four patients with protein S deficiency and in 10 patients with protein C deficit (Table 1). In one instance a combined protein C and protein S deficiency was present. In addition, at least six patients with FV Leiden polymorphism and one patient with the G20210A polymorphism were also described to have ICT (Table 2).

**Table 1:** Intracardiac Thrombosis Seen in Patients With Antithrombin, Heparin Cofactor II, Protein C and Protein S Deficiencies

Authors	Defect	Age, gender	Site of thrombosis	Associated risk factors	Therapy; evolution	Genotype	Comments
Gonzales – Levin (1984)	AT	55, M	Aortic porcine prothesis	LV failure	Replacement of prosthesis, UH, warfarin; fair	Het	
Santangeli et al (2008)	AT	47, F	LA	AF, diabetes type II, hypertension, high cholesterol	LMWH, warfarin, beta-blockers, statins; good	Het	Thrombus discovered 1 day after surgery for tibial fracture
Nagael et al (1990)	Hep co-factor II	14, F	RV	DVT	Surgery, streptokinase; fair	Het	Recurrence after 2 months with PE
Ozkutlu et al (1993)	Protein C	4, F	TV	None	Surgery; good	Hom	Father had also protein C deficiency
Letts R et al (1999)	Protein C	11, M	RA and DVT	Septic arthritis	Antibiotics, UH, warfarin; good	Het	
Nair et al (2001)	Protein C	43, M	LV	None	Surgery; good	Probably Het	Previous recurrent DV and PE
Matitian et al (2001)	Protein C	2, F	LV	None	Surgery, warfarin; good	Het	Brother with protein C deficiency but no cardiac thrombus
Saleh (2002)	Protein C	17, M	RA and LA	None	UH, warfarin; good	Het	
Keyikoioglu et al (2005)	Protein C	35, M	RV and TV	None	Lysis; good	Het	
Shindo et al (2006)	Protein C	30, M	AV	None	Surgery; good	Het	Non-coronary cusp was involved
Dogan et al (2006)	Protein C	42, F	RA and RV	Oral contraceptives	Surgery, discontinuation of oral contraceptives, ASA; good	Het	
Yokoyama et al (2009)	Protein C	76, M	AV	Old age	Surgery; good	Het	
Urgesi et al (2010)	Protein C	38, F	RV	Ulcerative colitis	LMWH; good	Het	PE also present
Girolami et al (1994)	Protein S	31, M	RA	None	UH, warfarin; fair	Het	Had peripheral embolism
Jobic et al (1999)	Protein S	77, F	AV	Old age	Surgery; good	Het	Thrombosis caused repeated embolic MI
Flora et al (2004)	Protein S	28, F	RV	Weak lupus anticoagulant	n.r.	n.r.	
Maldjian et al (2006)	Protein S	39, F	RA	None	Poor	Het	Patient had also PE with residual pulmonary hypertension
Aysemur et al (2007)	Combined Prot C and Prot S	3, F	RV and LV	None	UH, warfarin; good	Compound het	Brother also affected but without cardiac thrombosis

AT, antithrombin; LV, left ventricle; UH, unfractioned heparin; Het, heterozygous; LA, left atrium; AF, atrial fibrillation; LMWH, low molecular weight heparin; RV, right ventricle; DVT, deep vein thrombosis; PE, pulmonary embolism; TV, tricuspid valve; Hom, homozygous; RA, right atrium; AV, aortic valve; ASA, acetyl salicylic acid; MI, myocardial infarction.

**Table 2:** Reported Cases of Cardiac Thrombosis in Prothrombotic Polymorphisms

Authors	Defect	Age, gender	Site of thrombosis	Associated risk factors	Therapy and evolution	Genotype	Comments
Simioni et al (1997)	FV Leiden	55, F	RA	AF	Thrombectomy, MV substitution, warfrin; good	Hom	
Kohlhese et al (1998)	FV Leiden	14, M	MV	Elevated apolipoprotein (a)	Thrombectomy, atrial valve substitution, orgaran; poor	Het	Family history positive for thrombosis. Patient had also embolectomy for left femoral artery
Scheer et al (2006)	FV Leiden	2, F	LA	Respiratory tract infection, broad QRS tachycardia	LMWH, beta-blockers; fair	Het	Patient had also systemic embolism and tachycardia recurrencies
Mizia-Stec et al (2006)	FV Leiden	26, M	LV + PV	Churg-Strauss vasculitis	Cortisone, warfarin; fair	Het	
Ohlenfont et al (2007)	G20210A prothrombin	29, M	RV	None	Thrombectomy, warfarin; good	Hom	Post PE
Eweda et al (2010)	FV Leiden	25, F	RA	Compound Het for two MTHFR mutations (C677T and A1298C)	Thrombectomy; good	Het	Patient had also multiple PE
Yazicioglu et al (2011)	FV Leiden	32, F	LA	Invasive procedures for closure of ostium secundum	UH, tirofiban, warfarin; good	Hom	ASA and clopidogrel administered before invasive procedure

RA, right atrium; AF, atrial fibrillation; MV, mitral valve; Hom, homozygous; Het, heterozygous; LA, left atrium; LMWH, low molecular weight heparin; LV, left ventricle; PV, pulmonary valve; RV, right ventricle; PE, pulmonary embolism; MTHFR, methyltetrahydrofolate reductase; UH, unfractioned heparin; ASA, acetyl salicylic acid.

Age varied between two and 77 (mean 28.8). Nine were male and 19 were female. Fifteen of the 25 patients had variable associated risk factors, such as heart failure (one case), oral contraceptives (one case), septic arthritis (one case), ulcerative colitis (one case), lupus anticoagulant (one case), cardiac surgery (one case), high homocysteine levels due to a MTHFR mutation (one case), respiratory tract infection (one case), Churg-Strauss vasculitis (one case), DVT (one case). Altogether, no significant pattern was present. Old age and AF were present in only instances, each (Table 1).

The right atrium (RA) or the left atrium (LA) was involved in six and three instances, respectively. In one additional instance thrombi were present in both atria. One of the patients with RA thrombus had also a right ventricle (RV) thrombus. The RV or left ventricle (LV) was involved in three and two cases, respectively. In one patient with RV thrombosis, the tricuspid valve (TV) was also involved in the thrombotic process. In another instance both ventricles were affected. Aortic valves (AV) were involved in four cases (one prosthetic and three native). TV and mitral valve (MV) were affected in one instance, each. Pulmonary valve (PV) was affected only in one patient with Churg-Strauss vasculitis and heterozygous FV Leiden. The highest prevalence was observed for antithrombin and protein C deficiencies (Table 3). Seven of the 25 ICT involved the valves; of the remaining 18 cases, four could be considered mural and the remaining were free or floating. In one case a “thrombus in transit pattern” in right heart was present.

**Table 3:** Approximate Prevalence of Prothrombotic Defects in the General Healthy Population, Reported Cases of Intracardiac Thrombosis in These Conditions and Estimated Overall Incidence

Defect	Approximate prevalence (%)	Reported cases of cardiac thrombosis	Estimated approximate total cases	Comments
AT deficiency	0.02	2	10,000	
Protein C deficiency	0.3	11	3667	
Protein S deficiency	1.6	6	375	
Combined Prot C & Prot S	n.a.	1	n.a.	
Heparin co-factor II	n.a.	1	n.a.	
FV Leiden polymorphism	5	6	120	Figures are probably misleading due to limited search
Prothrombin G20210A polymorphism	3	1	33	Same as above

n.a., not available.

Therapeutic approach was varied. Surgical removal of thrombus was carried out in 11 instances. In another case a thrombotic prosthetic valve was replaced. The remaining cases were treated conservatively with unfractioned heparin (UH), low molecular weight heparin (LMWH) and warfarin. Fibrinolytic agents (streptokinase) were used in one patient.

Altogether evolution was fair or good. In only two patients evolution was listed as poor but no fatality was reported (Table 1, 2).

## Discussion

ICT has been associated both with cardiac diseases (dilatative cardiomyopathy, post-MI, heart failure, etc…) [1-3] and extracardiac disease (Behcet’s disease, antiphospholipid antibodies syndrome, cancer, etc…) [4] (Table 4). Autopsy studies have shown that the condition is more frequent than what could be judged on clinical grounds [4]. The present study is the first one to tackle the prevalence of ICT in congenital thrombophilia. Classification of intracardiac thrombi, usually based on imaging technique, distinguishes between mural and free or floating thrombi. The size is variable and the shape is usually round. A special variant is the thrombus in transit which represents a right side embolus migrating from RA to RV to result in a pulmonary embolism [14]. The pattern seen in thrombophilic patients does not seem to differ from the general behavior.

**Table 4:** Main Causes Of Cardiac Thrombi

Cardiac conditions
Catheter associated
Atrial fibrillation
Cardiomyopathy (dilated, Tako-Tsubo)
Heart failure
Myocardial infarction
Endomyocardial fibrosis
Heart valves defects

Extracardiac conditions
Behcet’s Disease
Cancer
Deep vein thrombosis (en route thrombi to the lungs)
Heparin-induced thrombocytopenia
Thrombophilic defects
Systemic lupus erytematosus
Amyloidosis

The modality of presentation of ICT is variable, it may represent a medical emergency but is may also be paucisymptomatic or asymptomatic. ICT in the congenital prothrombotic conditions may occur spontaneously or in association with other potential risk factors, safe for the presence of a recent MI which was a criterion for exclusion. There are reported cases of ICT in all conditions commonly recognized to be prothrombotic. The relatively low prevalence of ICT in patients with the prothrombin G20210A polymorphism as compared with FV Leiden may support the opinion of those who claim that this defect is a mild prothrombotic condition [30, 31]. Recent studies have indicated that homozygous patients with this polymorphism, contrary to what happens for heterozygotes, do not present an increased risk of thrombotic recurrences [32]. A paradox that remains unexplained and has no biological plausibility.

Since the prothrombin polymorphism is almost as prevalent as FV Leiden, it is surprising that only one case has been reported so far. However the possibility that the condition has not been adequately investigated cannot be ruled out.

The most striking observation of this paper is the high prevalence of ICT seen in patients with antithrombin, protein C and protein S deficiencies. These are surely the most frequently conditions involved. It is surprising also that protein C defect has been found in a higher number of patients than in protein S deficiency (Table 3). The reason for this discrepancy is unknown and inexplicable when one takes into account that protein S is a co-factor of protein C and that, therefore, their main biological effect is the neutralization of residual activated FV and FVIII. This is more so if one takes into account that the prevalence of protein S defect is at least three times as higher than that of protein C [33]. The difficulties commonly encountered in the classification of protein S defects due to the presence of both a combined and a free form, as compared with that of protein C deficiency may play a role. Another remarkable observation is the high prevalence of ICT in antithrombin deficiency, which is the rarest congenital prothrombotic condition. Since antithrombin deficiency is the most severe congenital prothrombotic condition, this could have been anticipated but was unrecognized.

We have excluded patients with known dilated cardiomyopathy. However it is worth noting that a prothrombotic defect (FV Leiden) has been demonstrated to be present in 12 out of 22 patients with LV thrombus and dilated cardiomyopathy but only in four of 38 patients without thrombus [34, 35]. The significance of this observation remains unclear since other studies have shown that FV Leiden and other polymorphisms played no role in the development of post-MI thrombus formation [36].

The role played by associated risk factors is not clear. Associated risk factors were present in 15 of the 25 patients. They were quite variable and sporadic. Only two conditions (old age and AF) were present in two patients. Because of this variability, no sure conclusions can be drawn on the role played by associated risk factors. The observation that the thrombotic event occurred often at young age (14 patients were less than 35) indicates the importance of the prothrombotic conditions. This is even more so if one takes into account that no cardiac abnormality was present in these young patients.

Cardiac thrombosis is a relatively rare condition in clinical practice, but the frequent presence of a prothrombotic defect, especially in antithrombin, protein C or protein S deficiency, in these patients indicates the need for a prothrombotic screening. This may supply useful information as to management of single cases and decrease mortality and secondary morbidity. Conversely, the unexpected relative elevated incidence of cardiac thrombosis found in these thrombophilic states indicates also the need for a thorough cardiac evaluation in all patients, including, in doubtful cases, even a transesophageal sonography. On the contrary, the low or very low prevalence seen in patients with polymorphisms which are very common (about 3-5% of population) suggests that echographic procedures are not needed.
